# Structural Basis for Certain Naturally Occurring Bioflavonoids to Function as Reducing Co-Substrates of Cyclooxygenase I and II

**DOI:** 10.1371/journal.pone.0012316

**Published:** 2010-08-23

**Authors:** Pan Wang, Hyoung-Woo Bai, Bao Ting Zhu

**Affiliations:** Department of Pharmacology, Toxicology and Therapeutics, School of Medicine, University of Kansas Medical Center, Kansas City, Kansas, United States of America; Baylor College of Medicine, United States of America

## Abstract

**Background:**

Recent studies showed that some of the dietary bioflavonoids can strongly stimulate the catalytic activity of cyclooxygenase (COX) I and II *in vitro* and *in vivo*, presumably by facilitating enzyme re-activation. In this study, we sought to understand the structural basis of COX activation by these dietary compounds.

**Methodology/Principal Findings:**

A combination of molecular modeling studies, biochemical analysis and site-directed mutagenesis assay was used as research tools. Three-dimensional quantitative structure-activity relationship analysis (QSAR/CoMFA) predicted that the ability of bioflavonoids to activate COX I and II depends heavily on their *B*-ring structure, a moiety known to be associated with strong antioxidant ability. Using the homology modeling and docking approaches, we identified the peroxidase active site of COX I and II as the binding site for bioflavonoids. Upon binding to this site, bioflavonoid can directly interact with hematin of the COX enzyme and facilitate the electron transfer from bioflavonoid to hematin. The docking results were verified by biochemical analysis, which reveals that when the cyclooxygenase activity of COXs is inhibited by covalent modification, myricetin can still stimulate the conversion of PGG_2_ to PGE_2_, a reaction selectively catalyzed by the peroxidase activity. Using the site-directed mutagenesis analysis, we confirmed that Q189 at the peroxidase site of COX II is essential for bioflavonoids to bind and re-activate its catalytic activity.

**Conclusions/Significance:**

These findings provide the structural basis for bioflavonoids to function as high-affinity reducing co-substrates of COXs through binding to the peroxidase active site, facilitating electron transfer and enzyme re-activation.

## Introduction

Cyclooxygenase (COX) I and II catalyze the metabolism of arachidonic acid (AA), resulting in the formation of prostaglandins (PGs), thromboxanes, and hydroxyeicosateraenoic acids (HETEs) [1]–[4], which exert an array of important biological actions in the body [5]–[7]. In humans, COX I and II share approximately 60% overall sequence similarity, and the sequence homology in their catalytic sites is even higher [8]–[10]. Whereas COX I is a constitutively-expressed enzyme in most tissues and primarily functions as a house-keeping enzyme, COX II is a highly-inducible enzyme (such as in the presence of various mitogens) and plays a critical role in mediating inflammation [11].

Both COX I and II have two catalytic activities that are functionally coupled: one is to catalyze the cyclooxygenase reaction that converts AA to prostaglandin G_2_ (PGG_2_), and the other one is to catalyzes the peroxidase reaction that reduces PGG_2_ to prostaglandin H_2_ (PGH_2_). These two reactions occur at distinct but functionally-related catalytic sites. A branched-chain model has been proposed to explain the mechanism of those two reactions catalyzed by COX I and II [12]. Based on this model, a peroxide (such as PGG_2_) is thought to initiate the peroxidase reaction by abstracting two electrons from hematin in the peroxidase active site, yielding *Compound I*, a protoporphyrin IX (PPIX) radical cation with an oxyferry group (Fe^4+^ = O). Next, *Compound I* undergoes an intra-molecular reduction by Tyr385 to form *Intermediate II* with a neutral PPIX and a Tyr385 tyrosyl radical. Alternatively, *Compound I* can undergo the one-electron reduction by an exogenous electron donor, yielding *Compound II* with a neutral PPIX and Tyr385. *Intermediate II* initiates the cyclooxygenase reaction by abstracting one hydrogen atom from arachidonic acid to yield an arachidonate radical, which then reacts with two molecular O_2_ to produce PGG_2_.

Suicide inactivation is a known phenomenon of the COX enzymes commonly observed during the catalysis of *in vitro* reactions, resulting in the loss of the peroxidase and cyclooxygenase activities in less than 1–2 min [13], [14]. Mechanistically, *Intermediate II* is thought to initiate the suicidal inactivation by forming *Intermediate III*, which causes further unknown protein modifications resulting in enzyme inactivation [13].

In recent studies [15], [16], we showed that some of the dietary bioflavonoids are strong activators of the catalytic activity of COX I and II *in vitro*, in cultured cells, and also *in vivo*. In this study, we sought to determine the structural basis and molecular mechanism by which these bioflavonoids activate COX enzymes. We jointly used the three-dimensional quantitative structure-activity relationship/comparative molecular field analysis (3-D-QSAR/CoMFA) and molecular docking approach, in combination with biochemical analysis and site-directed mutagenesis studies. We found that some of the bioflavonoids can bind tightly into the peroxidase active site and directly interact with the hematin component of the COX enzymes to facilitate the electron transfer from bioflavonoids to hematin. The findings of this study provide the mechanistic understanding and structural basis for bioflavonoids to function as high-affinity reducing co-substrates/activators of COXs by interacting with hematin for re-activation of the enzymes.

## Results

### QSAR/CoMFA analysis of the activation of the catalytic activity of COX I and II by bioflavonoids

**Figure 1:**
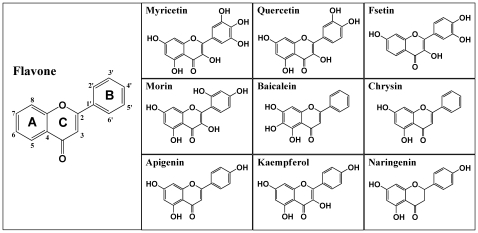
Chemical structures of the bioflavonoids used in this study. The structure of flavone is enlarged to show the numbering of different carbon positions.

**Figure 2:**
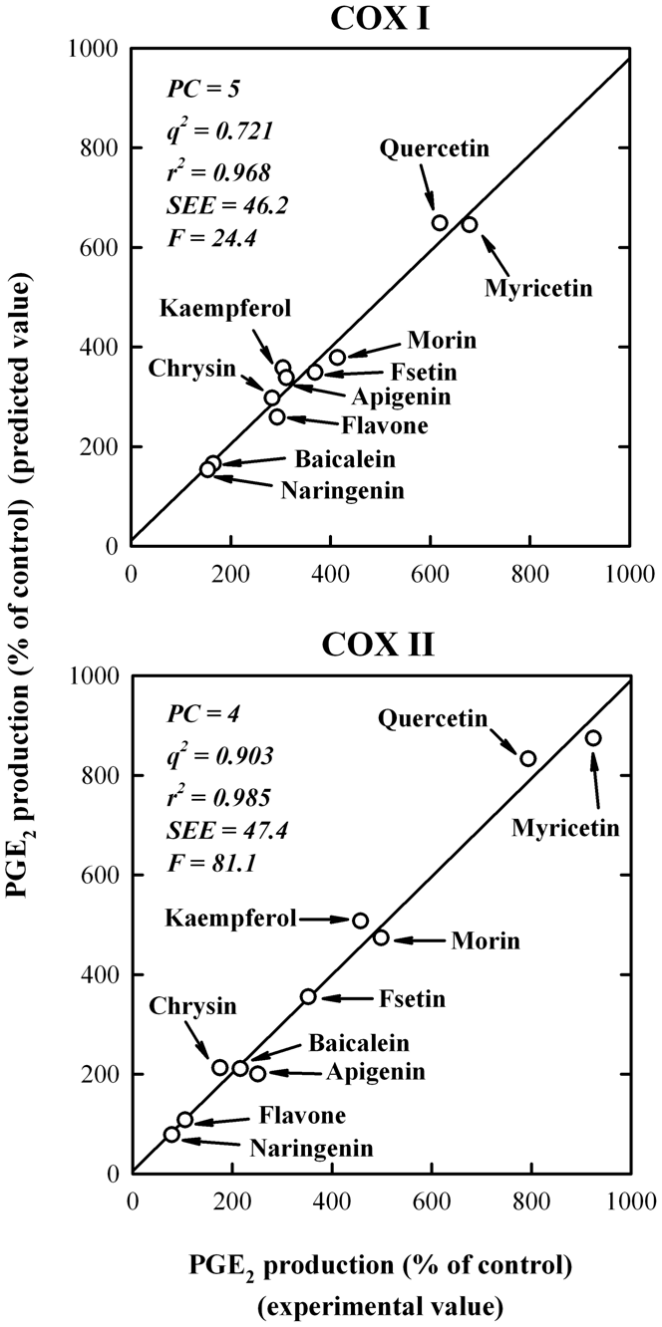
The 3-D QSAR/CoMFA analysis showing the correlation between the experimentally-determined COX-stimulating activity values and the predicted values. Nine representative bioflavonoids plus flavone were used in the analysis. The experimental values were based on measuring PGE_2_ production, which was determined in our previous study [15]. Statistical parameters (*q* ^2^, *r* ^2^, PC, SEE, and F) for the CoMFA models of COX I and II are also listed in the figure.

To probe the structural determinants of various bioflavonoids for activating COX I and II, we developed the 3-D QSAR/CoMFA models by using the experimental data obtained from 9 representative bioflavonoids (myricetin, quercetin, fisetin, morin, baicalein, chrysin, apigenin, kaempferol and naringenin) and flavone. These compounds were selected from our recent study [15], and they all share a highly-similar core structure (structures shown in **Figure 1**). In the QSAR/CoMFA analysis, the experimental values are the COX I- or COX II-mediated production of PGE_2_ (a major PG formed from AA) *in vitro* in the presence or absence of a dietary compound [15]. The relevant statistical parameters (PC, *q* ^2^, *r* ^2^, SEE, and F) for the CoMFA models of COX I and II are listed in **Figure 2**. The contributions from the steric and electrostatic fields were 42.5% and 57.5%, respectively, for the COX I CoMFA model, and 56.7% and 43.3%, respectively, for the COX II CoMFA model. There is a high degree of correlation between the experimentally-determined values and the predicted values (**Figure 2**), with *r* ^2^ of 0.968 and 0.985, respectively, for COX I and II. In addition, the *q* ^2^ values are 0.721 and 0.903, respectively. Since the *q* ^2^ values are far greater than 0.5, it suggests that the 3-D QSAR/CoMFA models developed in this study have a very high predictive ability.

**Figure 3:**
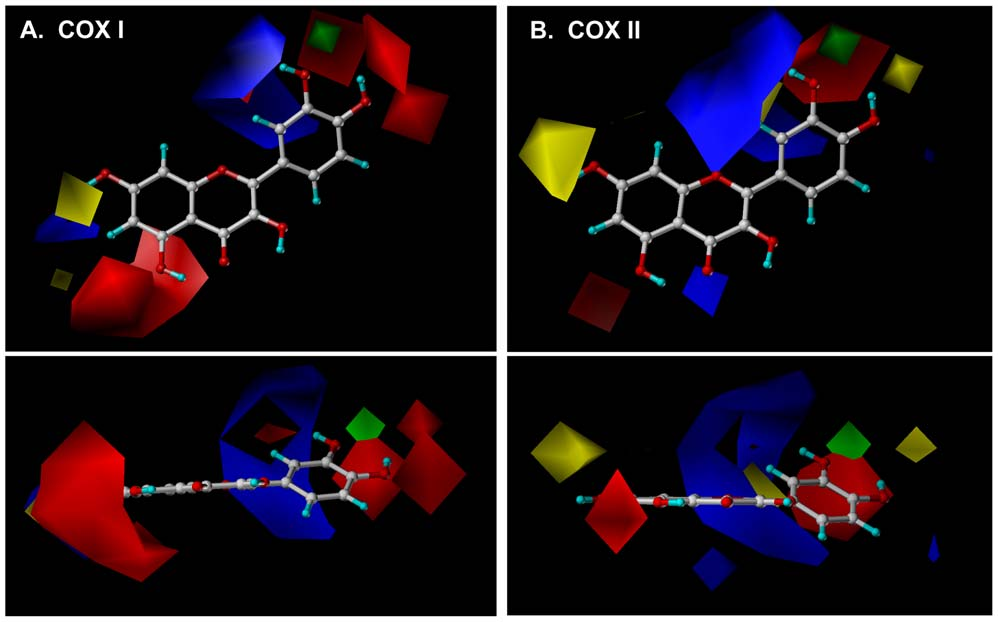
The 3-D QSAR/CoMFA color contour maps for COX I (A) and COX II (B). Note that quercetin was shown in the ball and stick format inside the field for demonstration. Oxygen, carbon, and hydrogen atoms are colored in red, gray, and blue, respectively. The contours of the steric maps are shown in yellow and green, and those of the electrostatic maps are shown in red and blue. Green contours indicate regions where a relatively bulkier substitution would increase the COX activity, whereas the yellow contours indicate areas where a bulkier substituent would decrease the COX activity. The red contours are regions where a negative-charged substitution likely would increase the COX activity whereas the blue contours showed areas where a negative-charged substitution would decrease the COX activity. Bioflavonoids with a higher ability to activate the COX enzymes are correlated with: (***i***) more bulkier substitute near green; (***ii***) less bulkier substitute near yellow; (***iii***) less negative charge near blue; and/or (***iv***) more negative charge near red. The figure in the lower panel shows the 90° rotation around the *x*-axis of the figure shown in the upper panel.

The contour maps derived from the CoMFA models for COX I and II are shown in **Figure 3**. Note that the quercetin molecule is depicted inside COX I and II for demonstration. The contours of the steric map are shown in *yellow* and *green*, and the contours of the electrostatic map are shown in *red* and *blue*. *Green* contours indicate regions where a steric bulkier substituent would increase COX activity, whereas the *yellow* contours would indicate areas where a steric bulkier substituent would decrease COX activity. The *red* contours indicate regions where a substituent with stronger negative charge would increase COX activity, whereas the *blue* contours showed areas where a substituent with stronger negative charge would decrease COX activity. There are a number of similarities between the contour maps for COX I and II. Firstly, the contour maps for both COX enzymes indicate the importance of the negatively-charged substitutes around the 3′, 4′ and 5′ positions of *B*-ring for activating the catalytic activity of the COX enzymes (**Figure 3**, red area). This suggestion is in agreement with the observed higher ability of some of the compounds with hydroxyl groups in their *B*-rings (such as myricetin) to stimulate the COX activity as compared to compounds without hydroxyl groups in their *B*-ring such as baicalein. In addition, the contour maps also suggest that the negatively-charged substitutes near the 2′ position of the *B*-ring may decrease the COX activity (**Figure 3**, blue area). This suggestion also agrees well with the higher stimulation of the COX activity by quercetin (with hydroxyl groups at the 3, 5, 7, 3′ and 4′ positions) compared to morin (with hydroxyl groups at 3, 5, 7, 2′ and 4′ positions). Furthermore, the contour maps predict that a bulker substitute near the 3′ position would increase the COX activity (**Figure 3**, green area).

### Molecular modeling analysis of the binding interactions of bioflavonoids with COX I and II

**Figure 4:**
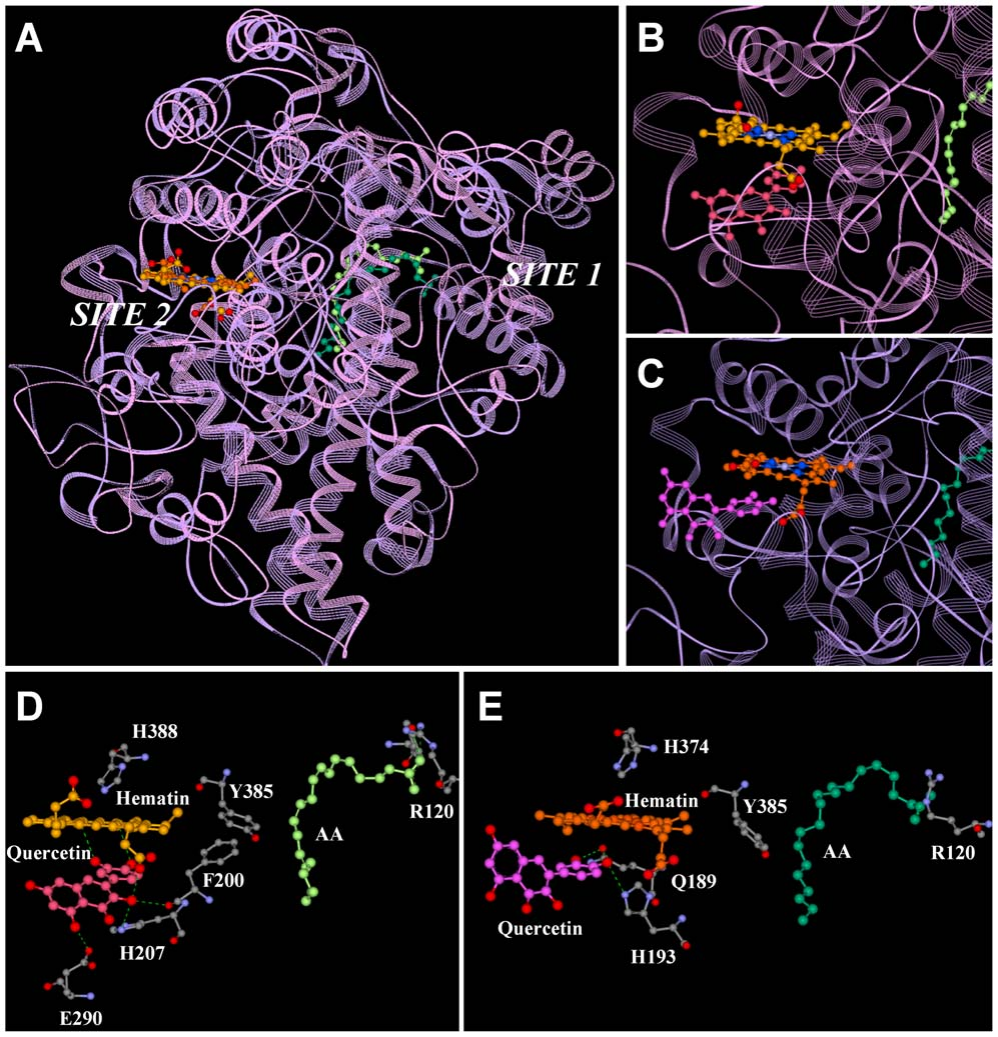
Identification of the binding sites for COX I and II by molecular docking approach. **A**. The superimposed structures of COX I and II in complex with hematin and AA. The white labels indicate the two potential binding sites for quercetin as identified by the *Active-Site-Search* function in *InsightII*. **B**. Docking results for quercetin in Site-2 of COX I. **C**. Docking results for quercetin in Site-2 of COX II. **D**. Enlarged view of the interaction of quercetin with hematin and key amino acid residues in the peroxidase active sites of COX I. **E**. Enlarged view of the interaction of quercetin with hematin and key amino acid residues in the peroxidase active site of COX II. The protein structure was shown in the ribbon format in **A**, **B** and **C**. COX I was colored in pink and COX II in purple. AA was colored in light green for COX I and dark green for COX II. Quercetin was colored in light red for COX I and magenta for COX II. Carbon atoms in hematin were colored in yellow for COX I and orange for COX II whereas nitrogen atoms were colored in blue, oxygen atoms in red, and magnesium in silver. The green dashes represent the hydrogen bonds. Hematin, AA, key amino acid residues, and quercetin are shown in the ball and stick format. For amino acid residues, oxygen atoms are shown in red, carbon atoms in gray, and nitrogen atoms in blue. Hydrogens are omitted in these molecules.

The 3-D structural models of the human COX I and II were successfully built using the *Modeler* program in *InsightII* according to the known *x*-ray structures of the sheep COX I (PDB code: 1diy) and mouse COX II (PDB code: 1dcx), respectively, as templates. As shown in **Figure 4A**, only two candidate binding sites (named as Site-1 and Site-2) were predicted by the *Active-Site-Search* function of the *Binding-Site* module in *InsightII*. Site-1 was located in the *N*-terminus of the COX proteins, which is part of the membrane-binding domain (imbedded inside the membrane). In reality, this site would not be accessible by the highly hydrophilic bioflavonoids. Site-2 is located close to the hematin structure and far away from the membrane, which is actually the known peroxidase catalytic site [3]. The *Simulated-Annealing* function in the *Docking* module was then used to dock quercetin into Site-2. The binding pocket was defined to include amino acid residues within a 4-Å reach around Site-2. One hundred docking modes were calculated and the conformation with the lowest energy value was then further minimized. **Figure 4B** and **4C** show the docked structures of quercetin in the peroxidase catalytic sites of COX I and II, respectively. Notably, quercetin was found to bind inside a rather deep binding pocket in both COX I and II, which is immediately next to hematin. The docking models predict that the binding site in COX I is relatively deeper than that in COX II, partly due to the different loop structures (composed of amino acid residues 181–190 for COX I and 167–176 for COX II) of these two enzymes.

Our recent study showed that quercetin and myricetin have a high potency for activating the COX activity in cultured cells, with an apparent *EC*_50_ value of approximately 50 nM [15]. As shown in **Figure 4D** and **4E**, several hydrogen bonds are formed between quercetin, hematin and the amino acid residues in the binding sites, which would be in line with its high binding affinity for the COX enzymes. For COX I, one hydrogen bond is formed between quercetin and hematin and four hydrogen bonds are formed between quercetin and amino acid residues E290, H207 and F200. For COX II, three hydrogen bonds are formed between quercetin and amino acid residues Q189 and H193.

By using the same method, we also docked myricetin, chrysin and flavone into the enzyme binding pockets of COX I and II. The binding energy values for all four ligands are summarized in **Table 1**. Notably, myricetin has one more hydroxyl group in its *B*-ring than does quercetin, and its binding energy values for COX I and II are lower than those of quercetin. This information led to the suggestion that myricetin has a higher binding affinity for COX I and II than quercetin, which agrees with our earlier study [15] showing that myricetin reached the stimulation plateau at lower concentrations than quercetin.

**Table 1:** The computed binding energy values (Δ*E*_binding_) for the molecular docking study for the binding of myricetin, quercetin, chrysin or flavone with human COX I or COX II.

Bioflavonoids	COX I Δ*E*_binding_ (kcal/mol)	COX II Δ*E*_binding_ (kcal/mol)
Myricetin	−109.23	−178.65
Quercetin	−83.50	−156.20
Chrysin	−40.33	−95.25
Flavone	−66.66	−88.32

The ligand-enzyme interaction energy value (Δ*E*_binding_) was calculated using the following equation: Δ*E*_binding_ = *E*_complex_−(*E*_COX_+*E*_ligand_), where *E*_complex_ was the potential energy for the complex of COX bound with the ligand, *E*_COX_ was the potential energy of the enzyme alone, and *E*_ligand_ was the potential energy for the ligand alone.

Notably, chrysin and flavone have no hydroxyl groups in their *B*-rings, but they could still bind to the same binding sites of the COX enzymes with relatively-lower binding affinities compared to myricetin and quercetin. Because of these structural and pharmacological features, it is predicted that these two bioflavonoids may be able to function as antagonists that would block the binding of the bioactive co-substrates (such as quercetin and myricetin) to the catalytic active sites and thus reduce their stimulation of the COX enzymes. This mechanistic explanation is supported by the observation made in our recent study [15] showing that flavone could suppress, in a concentration-dependent manner, the COX II-mediated formation of PGs in LPS-pretreated RAW cells [15].

### Experimental results in support of the docking models

#### Biochemical analysis

**Figure 5:**
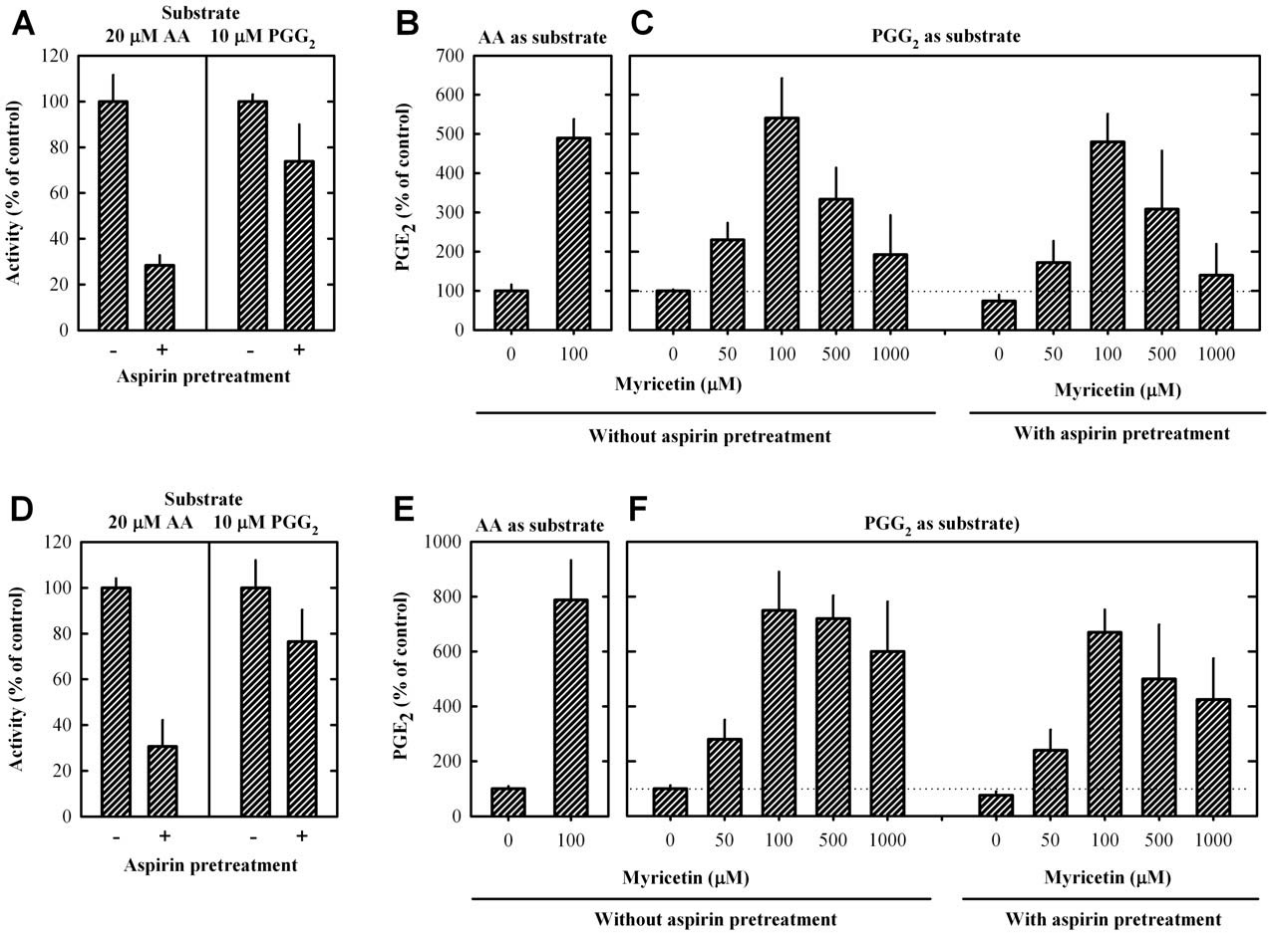
Myricetin stimulates the catalytic activity of COX I and II (with or without aspirin pretreatment) when [^14^C]AA or PGG_2_ is used as substrate. The incubation mixtures consisted of 20 µM [^14^C]AA (0.2 µCi) or 10 µM PGG_2_ as substrate, COX I or COX II as enzyme (0.5 or 0.97 µg/mL, respectively), 10 mM EDTA, 1 mM reduced glutathione, 1 µM hematin, and myricetin in 200 µL Tris-HCl buffer (100 mM, pH 7.4). The reaction was incubated at 37°C for 5 min and terminated by adding 15 µL of 0.5 N HCl to each test tube. Ethyl acetate (600 µL) was added immediately for extraction. The dried extracts were re-dissolved in acetonitrile or EIA buffer (Cayman Co. Michigan, USA), and the metabolites were analyzed using HPLC (with radioactivity detection) when [^14^C]AA was used as substrate [15] or using an EIA kit when PGG_2_ was used as substrate. Note that in this experiment, the COX I and II enzymes with or without aspirin pretreatment were both tested. For aspirin pretreatment, enzymes were pre-incubated with aspirin at 0.5 mM for COX I or 5 mM for COX II for 30 min at room temperature and then were immediately used as the enzyme source in the assay.

To provide experimental evidence for the computational model that bioflavonoids can stimulate the catalytic activity of COXs by binding to the peroxidase site but not the cyclooxygenase site, we studied the stimulatory effect of myricetin (a representative bioflavonoid) on the catalytic activity of COX I and II pretreated with aspirin, which can covalently acetylate and thereby inactivate the cyclooxygenase active site in these enzymes. To selectively test the effect of myricetin on the peroxidase activity of COX enzymes, PGG_2_ was used as a substrate to bypass the cyclooxygenation step (*i.e.*, the conversion of AA to PGG_2_). As shown in **Figure 5A** and **5D**, pretreatment of COX I and II with aspirin (0.5 and 5 mM, respectively) strongly inhibited the cyclooxygenase activity by 72% and 70%, respectively, when [^14^C]AA was used as substrate. However, when PGG_2_ was used as substrate, aspirin-pretreated COX I and II did not exert the same level of inhibition of their COX activity, which was as expected.

In the presence of myricetin, the catalytic activity of the control COX I and II (without aspirin pretreatment) was activated by 5- and 8-fold, respectively, when [^14^C]AA was the substrate (**Figure 5B** and **5E**). When PGG_2_ was used as substrate (as shown in **Figure 5C** and **5F**), myricetin stimulated the catalytic activity of COX I and II that were either pretreated with aspirin or received no pretreatment to a similar extent. Furthermore, the fold of stimulation was similar to what was seen with untreated COX I and II when [^14^C]AA was used as substrate (**Figure 5B** and **5E**). Taken together, these data clearly show that myricetin activates COX I and II through interaction with the peroxidase site but not the cyclooxygenase site. Notably, at higher concentrations of myricetin (500 and 1000 µM), the catalytic activity of COX I and II was inhibited to a similar degree, regardless of whether or not the enzymes were pretreated with aspirin. This additional observation indicates that the inhibition of the COX activity by high concentrations of bioflavonoids is due to the inhibition of the peroxidase catalytic activity.

#### Mutagenesis studies

To confirm the binding models obtained from our molecular docking studies showing that hydrogen bonds are formed between a bioflavonoid molecule and the amino acid residues Q189 and H193 of COX II (**Figure 4E**), we used the site-directed mutagenesis approach to verify the functional role of these two amino acid residues. We first made the Q189A, H193A and Q189A/H193A mutant proteins of COX II (expressed in cos-7 cells), and their levels of expression were confirmed using Western blot analysis (data not shown). As summarized in **Table 2**, the catalytic activity of the Q189A, H193A and Q189A/H193A mutant proteins was approximately 32%, 10.2% and 0% of the wild-type COX II activity. This observation showed that H193 played a more important role than Q189 in catalyzing the peroxidation reaction of the substrate. As shown in **Table 2**, myricetin could not stimulate the catalytic activity of the Q189A and H193A mutants, instead it inhibited their activity. Because the H193 mutant lost most of the catalytic activity, we chose to further study the interaction of Q189 with bioflavonoids by designing three additional mutant COX II proteins, *i.e.*, Q189E, Q189N and Q189R. Glutamic acid (E) is structurally very similar to glutamine (Q) with the same side-chain length and similar side-chain functional groups. Based on our docking model, we expected that the Q189E mutant likely would be able to bind bioflavonoids in a similar way as the wild-type COX II. In comparison, asparagine (N) has the same side-chain functional group but one carbon shorter than glutamine (Q), whereas arginine (R) has a positive-charged side chain compared with the relatively acidic side chain of glutamine (Q). As summarized in **Table 2**, Q189E and Q189N mutants retained approximately 80% and 56%, respectively, of the catalytic activity of the wild-type COX II, whereas Q189R retained approximately 30% of the catalytic activity. As we expected based on the docking models, myricetin could still stimulate the catalytic activity of the Q189E mutant by 51.4% over the corresponding control, but it did not stimulate the catalytic activity of the other two mutant enzymes (Q189N and Q189E), instead it inhibited their catalytic activity by 32.9% and 38.8%, respectively.

**Table 2:** The catalytic activity of several COX II mutants assayed in the presence or absence of myricetin.

Enzyme	COX II catalytic activity (formation of PGF_2α_ + PGE_2_ + PGD_2_; % of control)	
	Without myricetin	With 100 µM myricetin
Wild-type	100	224.5 (↑ 124.5%)^a^
Q189A	32.0	11.5 (↓ 74.1%)
H193A	10.2	N.D. (↓ 100%)
Q189A/H193A	N.D.^b^	N.D.
Q189E	78.5	118.9 (↑ 51.4%)
Q189N	55.9	37.3 (↓ 32.9%)
Q189R	37.9	23.2 (↓ 38.8%)

The catalytic activity of COX II enzyme was based on measuring the formation of PGF_2α_+PGE_2_+PGD_2_ from [^14^C]AA as substrate.

a The number in parenthesis represents the % of increase or decrease over the corresponding control (in the absence of myricetin).

b N.D., not detected.

## Discussion

In the present study, we conducted computational molecular modeling study and biochemical analysis to probe the mechanism of COX activation by dietary bioflavonoids. Data from the 3-D QSAR/CoMFA study predict that the *B*-ring of bioflavonoids play an important role for their direct activation of the catalytic activity of COX I and II. This notion is also supported by data from our docking studies, which show that bioflavonoids can bind to the peroxidase active site and directly interact with hematin, thereby facilitating the electron transfer from bioflavonoids to hematin. Additional biochemical analyses showed that when PGG_2_ is used as substrate, bioflavonoids can stimulate, to a similar degree, the catalytic activity of COX I and II with or without aspirin pretreatment of the enzymes. Furthermore, the site-directed mutagenesis analysis of the COX II enzyme confirmed the prediction of the docking model suggesting that Q189 in the peroxidase active site is important for the binding interaction with bioflavonoids and, subsequently, the stimulation of the COX II catalytic activity. Taken together, these data provide a rather detailed explanation of the molecular mechanism and structural basis for certain bioflavonoids to function as reducing co-substrates for the COX enzymes.

The structure-activity relationship regarding the antioxidant activity of various bioflavonoids has been extensively studied in the past [17]–[20]. Their antioxidant activity depends heavily on the number of phenolic hydroxyl groups as well as their localization in the molecules. The presence of a catechol structure (3′,4′-dihydroxyls) in the *B*-ring and a 2,3-double bond in the *C*-ring are important determinants for the high antioxidant activity [17]–[19]. The presence of 3- and 5-dihydroxyl groups usually further enhances the antioxidant activity. By comparing the stimulation of COX activity by apigenin and naringenin (**Figure 2**), we can see that the 2,3-double bond in the *C*-ring is an essential structure for bioflavonoids to have a stimulatory effect on the COX activity, although this point was not suggested by our CoMFA contour maps. The considerable similarity noted between our 3-D QSAR/CoMFA models and the earlier observations on the antioxidant activity of various bioflavonoids suggests that the ability of various bioflavonoids to directly stimulate the COX catalytic activity is, in a large part, attributable to their antioxidant property.

The molecular docking studies identified that bioflavonoids can bind to the peroxidase sites of COX I and II, thus suggesting the possibility that bioflavonoids stimulate their catalytic activity by interacting with the peroxidase sites. This suggestion was confirmed by biochemical analysis using PGG_2_ as substrate to bypass the cyclooxygenase reaction. Our observation that myricetin can stimulate the catalytic activity of COX enzymes to a similar degree with either PGG_2_ or AA as a substrate provides support for the notion that myricetin selectively activates the peroxidase activity. Our additional observation showing the lack of an effect of the aspirin pretreatment of the COX enzymes on myricetin's ability to activate their peroxidase activity (assayed using PGG_2_ as substrate) provides definitive evidence for this suggestion.

Our findings from our homology modeling and molecular docking studies provide detailed insights on the molecular mechanism of actions. As shown in **Figure 4**, bioflavonoids can fit into the peroxidase sites of COX I and II tightly and form several hydrogen bonds with the hematin moiety as well as with the nearby amino acid residues (in the case of quercetin, E290, H207 and F200 for COX I; H193 and Q189 for COX II). The formation of these hydrogen bonds facilitates the electron transfer from the *B*-ring of a bioflavonoid molecule to the hematin element of the enzymes. The binding energy values calculated from the docking models correlate well with the ability of the active bioflavonoids to stimulate the catalytic activity of the COX enzymes, which provides additional support for the docking models.

In addition, the docking model of COX II is also supported by the results of our site-directed mutagenesis studies. By mutating Q189 and H193 into alanine (A), we showed that Q189 and H193 are important for COX II activity, especially the peroxidase activity, which is consistent with an earlier report [21]. H193 plays a more important role than Q189 since the H193A mutant nearly loses all catalytic activity compared to the Q189A mutant. The observation that myricetin cannot activate the Q189A, Q189N, or Q189R mutant COX II enzyme revealed that the side chain of Q189 is vitally important for the binding interaction with bioflavonoids and also for their stimulation of the COX II activity. This experimental observation confirms our docking results showing that Q189 forms two hydrogen bonds with bioflavonoids (**Figure 4E**). As expected, the Q189E mutant can still be stimulated by myricetin because the side chain of glutamic acid (E) is of the same length and similar chemical property as the side chain of glutamine (Q), and thus hydrogen bonds can still be formed between glutamic acid and bioflavonoids. Although approximately 56% of the catalytic activity is retained in the Q189N mutant protein, myricetin cannot stimulate its catalytic activity, which indicates that the length of the side chain of Q189 is crucial for binding interactions with bioflavonoids.

**Figure 6:**
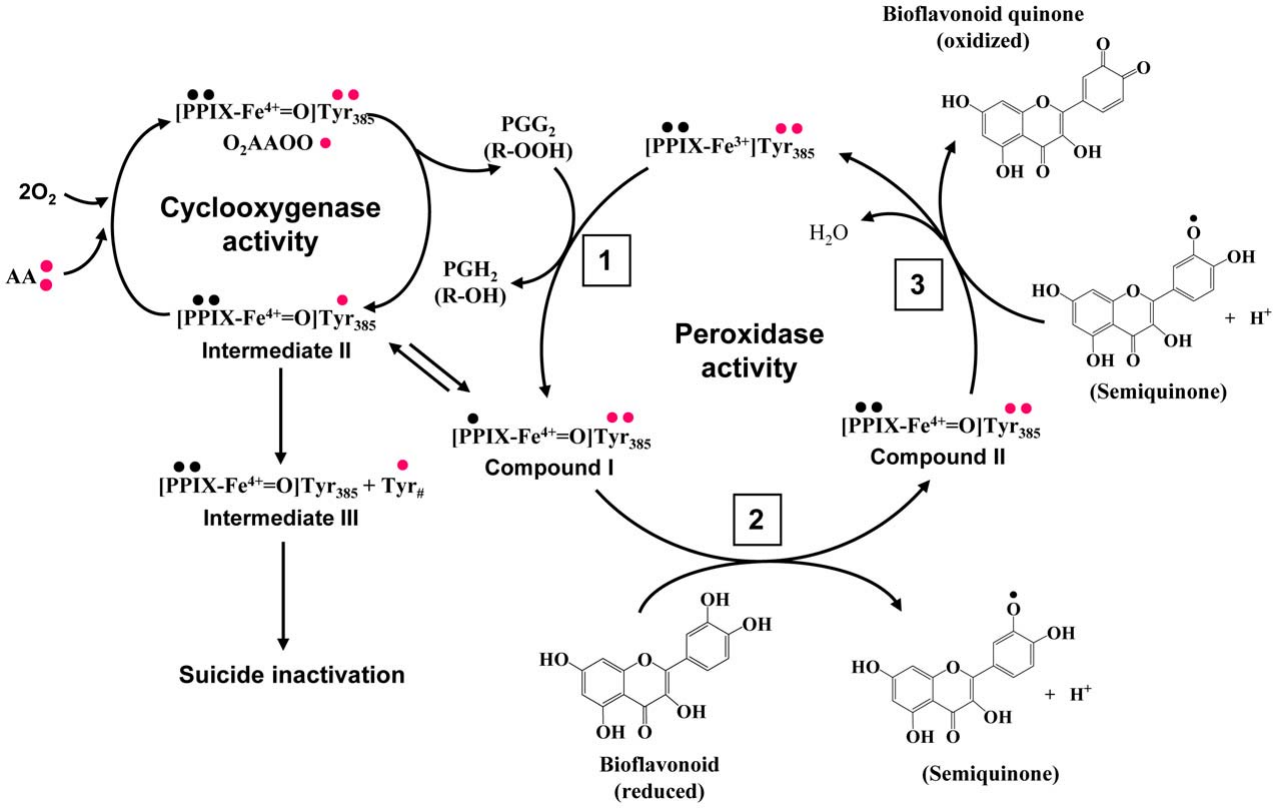
Schematic depiction of the catalysis and inactivation mechanism of COX enzymes and their interaction with bioflavonoids. PPIX is for protoporphorin IX. Quercetin structure is shown as a representative bioactive bioflavonoid. Events in the peroxidase cycle are labeled with numbers to denote the sequence of occurrence.

There were several earlier studies that investigated a number of reducing co-substrates of COXs [22]–[26]. The mechanism is generally thought to be due to the reduction of the oxidized intermediates by the co-substrates [23], [25]. As depicted in **Figure 6**, the reducing potential of bioflavonoids, like other reducing co-substrates, will help maintain the peroxidase cycle and thereby slow down the suicidal inactivation of the COX enzymes by donating one electron each to *Compound I* and *II* to restore the reducing activity of hematin, which is needed for the peroxidase to convert PGG_2_ to PGH_2_. The oxidized quinone form of bioflavonoids is expected to have a lower binding affinity for the peroxidase site because they will lose two hydrogen bond donors (hydroxyl groups), and some of the hydrogen bonds cannot be formed between the quinine form and the peroxidase site. Accordingly, the following catalytic sequence is proposed (depicted in **Figure 6**): It is assumed that PGG_2_ has a high binding affinity for the peroxidase site of the enzyme and will tightly bind to this site. Immediately following the catalytic conversion of PGG_2_ to its product, the product will dissociate from the enzyme (due to a reduced binding affinity). After that, the peroxidase site will become catalytically inactive (with an oxidized hematin), and it will be bound by a bioflavonoid molecule (in its reduced form) for the reduction of hematin to its initial state. During the process, the bioflavonoid is oxidized initially to semiquione (as an intermediate) and then to quinone. The bioflavonoid quinone will then be released from the activated peroxidase site because the oxidized molecule will have a reduced binding affinity for the peroxidase active site. In this model, it is apparent that there is a potential competition between the substrate (PGG_2_) and the co-substrate (bioflavonoid) at the peroxidase catalytic site. When the bioflavonoid concentration becomes too high, it will increase the fraction of the active peroxidase site that is still occupied by the reducing co-substrate, and when this occurs, it would inhibit the binding of PGG_2_ to the peroxidase site and thus would reduce the catalytic activity of the enzyme for the formation of further products. This mechanistic explanation is in agreement with the data shown in **Figure 5C** and **5F** as well as a number of earlier studies showing a concentration-dependent biphasic modulation of the COX activity by co-substrates, namely, the presence of a co-substrate at low concentrations stimulated the COX activity, whereas its presence at higher concentrations inhibited the COX activity [22]–[28]. Notably, some of the earlier studies suggested that the inhibition by high concentrations of the co-substrate was due to the fast reduction of *Intermediate II* and the loss of cyclooxygenase activity [27]. This explanation appears to disagree with the observation (in **Figure 5C** and **5F**) made in this study which showed that myricetin at high concentrations (500 and 1000 µM) inhibited the peroxidase activity of aspirin-pretreated COX II (using PGG_2_ as substrate) to a similar degree as they inhibited the catalytic activity of untreated COX II enzyme for its metabolism of AA as substrate.

In conclusion, the results of our present study show that some of the bioflavonoids can bind tightly to the peroxidase active site of the COX enzymes, and through direct interaction with hematin, the electrons are transferred from the bioflavonoid molecule to hematin, ultimately facilitating the re-activation of the COX enzymes during a catalytic cycle. The ability of bioflavonoids to re-activate the COX catalytic activity depends heavily on the structural features of their *B*-rings and their overall antioxidant activity.

## Materials and Methods

### Chemicals and reagents

[^14^C]Arachidonic acid ([^14^C]AA, specific radioactivity of 53 Ci/mol) was purchased from PerkinElmer (Boston, USA). Myricetin, aspirin, Dulbecco's modified Eagle's medium (DMEM) and fetal bovine serum (FBS) were purchased from Sigma-Aldrich Co. (St. Louis, MO). Human COX I and II, PGG_2_, and enzymatic immunoassay (EIA) kit for PGE_2_ were purchased from Cayman (Ann Arbor, Michigan). According to the supplier, the COX I and II preparations used in this study had a purity of approximately 95% and 70%, respectively. The cDNA of the human COX II was purchased from Origene Technologies (Rockville, MD). QuikChange XL site-directed mutagenesis kit and XL10-Gold competent cells were purchased from Stratagene (La Jolla, CA). QIAprep miniprep kit was obtained from Qiagen (Valencia, CA). Lipofectamine-2000 and goat anti-rabbit IgG conjugated to horseradish peroxidase were purchased form Invitrogen Co. (Carlsbad, CA). Polyclonal rabbit antibody against COX II was purchased from Cell Signaling Technology, Inc. (Danvers, MA). Cos-7 cells were obtained from the American Type Culture Collection (ATCC, Manassas, VA). The ECL Plus kit was purchased from GE Healthcare Bio-Sciences Corp. (Piscataway, NJ).

### Quantitative structure-activity relationship (QSAR) analysis

All calculations described in this study were carried out using the *SYBYL* molecular modeling program (V7.1, Tripos Inc., St. Louis, MO) installed in a Red Hat Enterprise Linux WS4.0 (Red Hat Inc. Raleigh, NC) operating system on a Dell Precision 690 workstation.

#### Molecular models and structural alignment

The chemicals used in this study comprised of a total of nine bioflavonoids (myricetin, quercetin, fisetin, morin, baicalein, chrysin, apigenin, kaempferol, and naringenin) plus flavone (structures shown in **Figure 1**). All molecules were constructed using the building tools of the *SYBYL7.1* molecular modeling software. The geometry of each molecule was optimized using the standard *Tripos* force field with the conjugate-gradient minimization to an energy change convergence criterion of 0.001 kcal/mol. All atomic partial charges were computed using the *Gasteiger-Marsili* method. All molecules were aligned to flavone as a template by using the rigid-body least-squares fitting method. After alignment, the molecules were placed in a 3-D cubic lattice with 2-Å spacing. If the calculated steric and electrostatic energy values were >30 kcal/mol, they were truncated to this value, an empirical approach that has been adopted in many similar studies [29].

#### 3-D QSAR/CoMFA analysis

For 3-D QSAR/CoMFA analysis, the method of partial least squares (PLS) regression was used to analyze the compounds by correlating the experimentally-determined COX activity and the computed values according to the CoMFA fields. The experimental values were adopted from our recent study [15], and they refer to the fold of stimulation of PGE_2_ production *in vitro* when 200 µM of a given flavonoid was present. In this study, we used one representative concentration (200 µM) of each bioflavonoid because our earlier analysis of concentration dependence showed that myricetin, quercetin, fisetin and morin (representative bioflavonoids tested) exerted nearly the peak COX stimulation for the production of PGE_2_ at this concentration [15]. Accordingly, the magnitude of peak stimulation was adopted to reflect the relative stimulatory efficacy/capacity of each bioflavonoid. In addition, we showed that when [^14^C]AA was used as substrate, the production of PGF_2α_, PGE_2_, PGD_2_ and 12-HHT was stimulated almost in a parallel manner when different concentrations of these bioflavonoids were present [15]. Based on these observations, we thus chose to use the formation of PGE_2_ as a representative product to conveniently reflect the COX catalytic activity *in vitro*.

The first PLS analysis was done to determine the optimum number of the principal components (PCs) by using the leave-one-out cross-validation procedure. In this method, each compound was systematically excluded once from the data set, after which its activity was predicted by a model derived from the remaining compounds. The *q* ^2^ value (*i.e.*, the cross-validated correlation coefficient value) was calculated based on these predictions in the first PLS analysis, and this parameter generally assesses a model's predictive ability. By setting the number of PCs to the optimum number, the second PLS analysis was carried out without cross-validation to calculate the correlation coefficient *r* ^2^. The *r* ^2^ generally reflects a model's overall goodness of fit to the experimentally-determined values for the compounds. In most studies, a model with *r* ^2^>0.9 and *q* ^2^>0.5 is generally considered to be of acceptable predictive ability.

### Molecular docking of bioflavonoids into the binding pockets of COX I and II

Energy minimization and molecular docking were performed on a Dell Precision 690 workstation using the *InsightII* modeling software (Version 2005, Accelrys Inc. San Diego, CA) installed in a Red Hat Enterprise Linux WS4.0 operating system. The *CVFF* force field was used for energy minimization with *Polak* and *Ribiere* conjuate gradients until the final convergence criterion reached the 0.001 kcal/molÅ. The structures of quercetin, myricetin and chrysin were built with the *Builder* module in *InsightII* and minimized with the *Discover* module.

#### Homology modeling

Since the *x*-ray structures of human COX I and II are not available at present, the sheep COX I structure (PDB code: 1diy [30]) and mouse COX II model (PDB code: 1dcx [31]) in complex with AA were used as templates to build the homology models of human COX I and II. Protein sequence analysis showed that the similarity between the sheep COX I (GI: 57164169) and human COX I (GI: 243972) is 93.85%, and the similarity between the mouse COX II (GI: 31981525) and human COX II (GI: 38565065) is 86.76%. Given the high degrees of their sequence similarity, we thus directly used the *Modeler* module of *InsightII* to generate five 3-D structure models for both human COX I and II. The models for COX I and II with the optimal side chain conformation and lowest potential energy were chosen for further minimization. Notably, because the hematin structure was missing in the mouse COX II (PDB code: 1dcx) model, another *x*-ray structure of the mouse COX II (PDB code: 5cox [32]) was used for superimposition with the 1dcx structure, and the precise positioning of hematin in the 5cox structure was adopted to the 1dcx structure and the whole structure was further minimized using *Molecular Dynamics* (a module in *InsightII Discover 3*) with the backbone of the protein held fixed.

#### Binding site determination and molecular docking

The binding sites on the COX enzymes for bioflavonoids were determined by using the *Active-Site-Search* function in the *Binding-Site* module of *InsightII*. The s*ite-open-size* parameter was set at 7 Å and the s*ite-cut-off-size* parameter was set at 420 Å^3^ based on the size of quercetin (12 Å×5 Å×7 Å). We defined the binding pocket with amino acid residues within 4 Å around the candidate binding sites. *Simulated Annealing* docking method in the *Affinity* module was used to dock each bioflavonoid molecule into the candidate binding pockets. One hundred docking modes were calculated and the ones with the lowest binding energy were chosen for further minimization. The interaction energy (Δ*E*_binding_) value between a given compound and the enzyme was calculated by using the following equation: Δ*E*_binding_ = *E*_complex_−(*E*_COX_+*E*_ligand_), where *E*_complex_ was the potential energy for the complex of COX bound with the ligand, *E*_COX_ was the potential energy of the enzyme alone, and *E*_ligand_ was the potential energy for the ligand alone. The interaction energy was used to estimate the relative interaction affinity between the enzyme and the dietary compound.

### Selective inhibition of the cyclooxygenase activity of COX I and II

Earlier studies showed that aspirin can selectively and covalently modify the cyclooxygenase active site (but not the peroxidase activity site), resulting in a selective knockout of the cyclooxygenase activity of the COX enzymes [33]–[35]. This approach was adopted in the present study, and the COX I and II enzymes were pre-incubated with aspirin (at 0.5 and 5 mM, respectively) for 30 min at room temperature.

For measuring the catalytic activity of untreated control COX I or COX II, ^14^C-AA was used as substrate, but for measuring the catalytic activity of aspirin-pretreated COX I or COX II, PGG_2_ was used as substrate. The reason that we used PGG_2_ as a substrate for the aspirin-pretreated COXs was because it is the intermediate product of AA formed by the cyclooxygenase activity of COXs, and it can still be further converted to form PGH_2_ by the peroxidase activity of the COXs. PGH_2_ can be further converted non-enzymatically to PGF_2α_, PGE_2_, and PGD_2_. Although the decomposition may be differentially affected by the presence of myricetin, our recent study [15] showed that when [^14^C]AA was used as substrate, the production of PGF_2α_, PGE_2_ and PGD_2_ catalyzed by COX I or COX II was stimulated almost in a parallel manner when different concentrations of myricetin were present. Therefore, measuring the changes in the sum of these PG products or in PGE_2_ alone was used as indicators of the changes in PGH_2_ level or the peroxidase activity of COXs.

Experimentally, the incubation mixtures (in Eppendrof tubes) included the following: 20 µM ^14^C-AA (0.2 µCi) or 10 µM PGG_2_ as substrate, COX I or COX II as the enzyme (0.5 or 0.97 µg/mL, respectively), 10 mM EDTA, 1 mM reduced glutathione, 1 µM hematin, and myricetin in a final volume of 200 µL Tris-HCl buffer (100 mM, pH 7.4). Myricetin was initially dissolved in pure ethanol as stock and then further diluted with the reaction buffer. The reaction was incubated at 37°C for 5 min and terminated by adding 15 µL of 0.5 N HCl to each tube. Ethyl acetate (600 µL) was added immediately for extraction. When [^14^C]AA was used as substrate, the dried extracts were re-dissolved in acetonitrile for analysis of representative PG products by HPLC, and when PGG_2_ was used as substrate, the dried extracts were re-dissolved in the EIA buffer for analysis of PGE_2_ using an EIA kit.

Since PGG_2_ may spontaneously decompose to PGH_2_ and then to other PGs (such as PGE_2_), here we also assessed the stability of PGG_2_ under the above *in vitro* reaction conditions. We incubated PGG_2_ at 37°C for 5 min in the same reaction buffer as described above (in the absence of the COX enzyme), and we found that less than 0.01% of PGG_2_ was spontaneously converted to PGE_2_ after a 5-min incubation at 37°C. Also, the presence of 100 µM myricetin under the same conditions did not significantly increase the spontaneous conversion of PGG_2_ to PGE_2_.

### *In vitro* site-directed mutagenesis study

The human COX II cDNA was used as template to generate the corresponding mutant cDNAs used in this study. The site-directed mutagenesis of the COX II gene *in vitro* was performed using a PCR-based QuikChange XL site-directed mutagenesis kit according to the procedures recommended by the manufacturers. The primers used for site-directed mutagenesis are listed in **Table 3**. The reconstructed plasmids were purified from the transformed XL10-Gold using the QIAprep Miniprep kit. Sequences of all reconstructed plasmid DNAs were confirmed by DNA sequencing. Cos-7 cells were maintained in DMEM supplemented with 10% FBS and used to express the wild-type and mutant COX II proteins. The transfection of reconstructed mutant plasmids was carried out using Lipofectamine-2000 according to the manufacturers' instructions.

**Table 3:** Primers used in the site-directed mutagenesis study.

COX II mutants	Primers	
Q189A	Forward Reverse	GCATTCTTTG CCGCGCACTT CACGCATCAG CTGATGCGTG AAGTGCGCGG CAAAGAATGC
H193A	Forward Reverse	GCCCAGCACT TCACGGCTCA GTTTTTCAAG CTTGAAAAAC TGAGCCGTGA AGTGCTGGGC
Q189A/H193A	Forward Reverse	ATTCTTTGCC GCGCACTTCA CGGCTCAGTT TTTC GAAAAACTGA GCCGTGAAGT GCGCGGCAAA GAAT
Q189N	Forward Reverse	GCATTCTTTG CCAATCACTT CACGCATCAG CTGATGCGTG AAGTGATTGG CAAAGAATGC
Q189R	Forward Reverse	GCATTCTTTG CCCGGCACTT CACGCATCAG CTGATGCGTG AAGTGCCGGG CAAAGAATGC
Q189E	Forward Reverse	GCATTCTTTG CCGAGCACTT CACGCATCAG CTGATGCGTG AAGTGCTCGG CAAAGAATGC

The sequences that are changed for each of the mutant COX proteins developed in this study are marked with underlines.

Thirty h after transfection with the plasmids, cells were collected by centrifugation and were then sonicated in ice-cold lysis buffer (50 mM Tris-HCl and 200 mM NaCl, pH 7.5). After addition of 5 mM 1,4-dithiothreitol and 1 mM phenylmethylsulfonyl fluoride (PMSF) to the crude homogenates, they were centrifuged at 10,000×g for 10 min at 4°C. After addition of 10% glycerol, the supernatants were stored at −80°C until they were used as enzyme source for assaying the COX II's catalytic activity. Western blot analysis was used to determine the expression of the wild-type and mutant human COX II proteins, which were separated by using the 10% SDS-polyacrylamide gel (SDS-PAGE) in a Mini-Protein system (BioRad, Hercules, CA). After electrophoresis, the protein bands on the gel were transferred onto PVDF membrane (BioRad, Hercules, CA). The membrane was first blocked with 5% non-fat dried milk powder in Tris-HCl-buffered saline containing 0.1% Tween-20 (the blocking solution), and then probed with polyclonal rabbit antibodies against COX II. The primary antibody-antigen complexes were detected using the goat anti-rabbit IgG conjugated to horseradish peroxidase and developed by using the ECL Plus kit.

The COX catalytic activity was assayed as described above, by using the whole cell lysates as the enzyme source and [^14^C]AA as the substrate. Measuring the combined formation of PGF_2α_, PGE_2_ and PGD_2_ was used to reflect the COX II catalytic activity.
